# Supplementary material for: The interactome of intact mitochondria by cross-linking mass spectrometry provides evidence for coexisting respiratory supercomplexes
Source: Mol Cell Proteomics. 2017 Dec 8;17(2):216–32. doi: 10.1074/mcp.RA117.000470 (PMC5795388; doi:10.1074/mcp.RA117.000470)
Supplement: Supplemental Data [file supp_RA117.000470_133922_0_supp_23978_fzffwf.zip › spectra_annotation/mito_spectra_annotation/451-1-10-1-5-1.pdf]

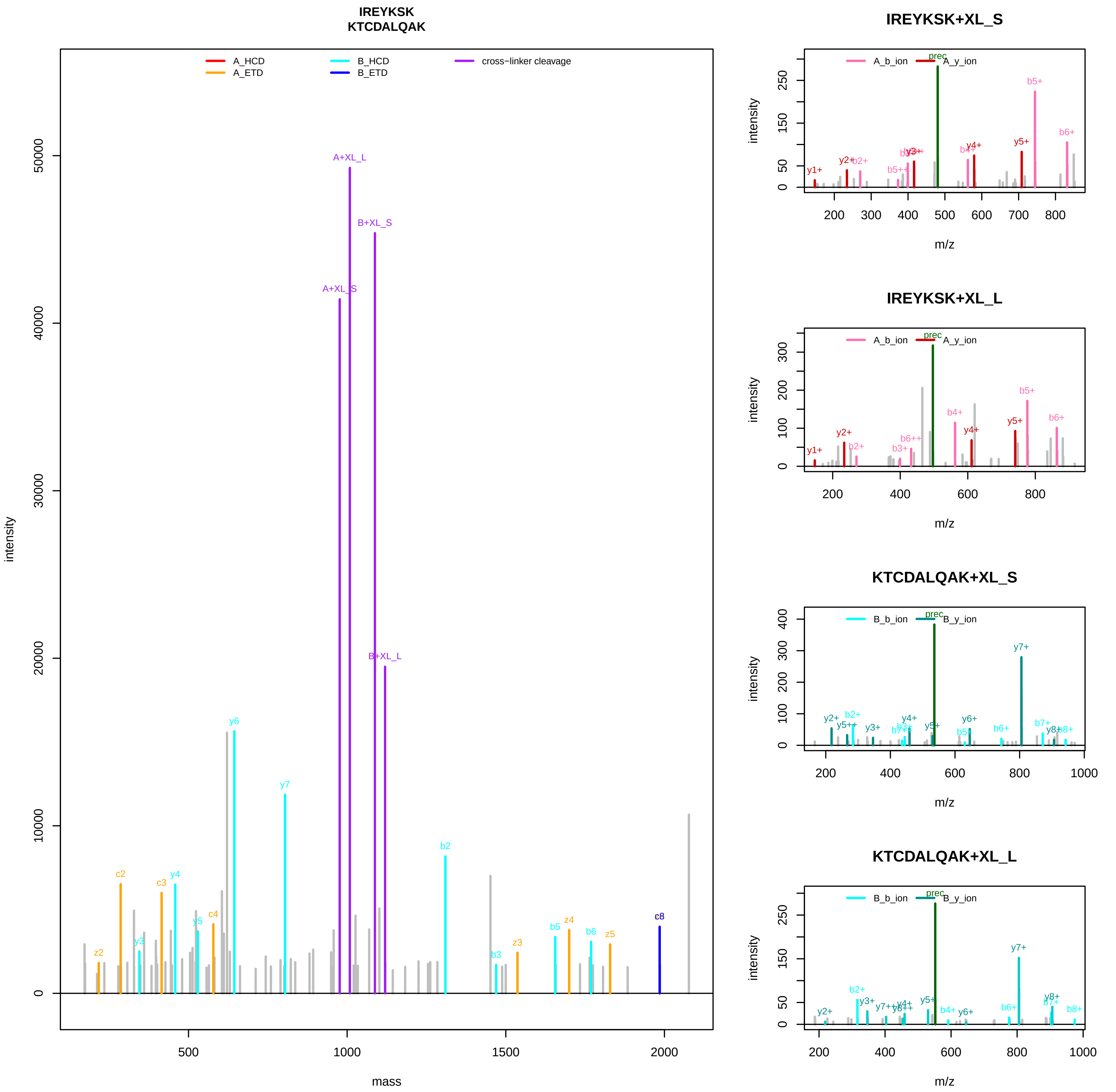
IREYKSK
KTCDALQAK
A_HCD
A_ETD
B_HCD
B_ETD
cross−linker cleavage
A+XL_L
B+XL_S
A+XL_S
B+XL_L
z2
c2
y3
c3
y4
y5
c4
y6
y7
b2
b3
z3
b5
z4
b6
z5
c8
intensity
mass
IREYKSK+XL_S
IREYKSK+XL_L
KTCDALQAK+XL_S
KTCDALQAK+XL_L
A_b_ion
A_y_ion
B_b_ion
B_y_ion
prec
m/z
y1+
y2+
b2+
b3+
b4+
b5+
b6+
y3+
y4+
y5+
b5++
b6++
y6+
y7+
y8+
b7+
b8+
y5++
y7++
y8++
